# 

**Published:** 1820-12

**Authors:** 


THE LONDON

### Medical and Physical Journal.

6 OF VOL. XLIV.] DECEMBER, 1820. [NO. 262.

NOTICE.

*The Proprietors beg to acquaint the Medical Profession, that the* GENERAL INDEX *is published, comprising an Analytical Table of Contents, from* VOLUME ONE TO FORTY *inclusive; carefully arranged in Alphabetical Order, with References to the whole of the cited Authorities, under their nominal Characters, &c. and forming an indispensable* APPENDIX *to the Journal; in one large* 8vo. *volume price* 21s.

### MONTHLY CATALOGUE OF MEDICAL BOOKS.

An Historical and Practical Treatise on the Internal Use of the Hydro-cyanic (Prussic) Acid in Pulmonary Consumption, and other Diseases of the Chest; as well as in several Complaints attended by great Nervous Irritation, or acute Pain; with full Directions for the Preparation and Administration of that Medicine; and a preliminary descriptive Account of the principal Diseases in which it has been employed, illustrated by numerous Cases. By A. B. Granville, M.D. F.R.S. F.L.S. M.R.I. Physician in ordinary to his Royal Highness the Duke of Clarence; Member of the Royal College of Physicians in London, &c. 12mo.

A Synopsis of the Diseases of the Eye, and their Treatment. To which are prefixed, a short Anatomical Description and a Sketch of the Physiology of that Organ. By Benjamin Travers, F.R.S. Surgeon to St. Thomas's Hospital. With Plates, coloured, 1l. 5s. 8vo.

A Practical Treatise on the Diseases of the Eye. By John Vetch, M.D. F.R.S. With Plates, coloured, 10s. 6d. 8vo.

Introductory Lecture, delivered in the Theatre of the Royal College of Surgeons, on the 8th of May, 1820. By B. C. Brodie, F.R.S. 8vo. 3s.

General Elements of Pathology. By Whitlock Nicholl, M.D. &c. 8vo. 9s.

A Descriptive, Diagnostic, and Practical Essay on Disorders of the Digestive Organs and general Health; and particularly on their numerous Forms and Complications, contrasted with some acute and insidious Diseases. Second Edition. By Marshall Hall, M.D. F.R.S. 8vo. 7s.

Illustrations of the capital Operations of Surgery. By Charles Bell. Large 4to. Parts I. and II. Coloured, 1l. 1s. each; plain, 15s.

Historic Sketch of the Causes, Progress, and Mortality, of the Contagious Fever epidemic in Ireland, during the years 1817, 18, and 19. 8vo. 16s.

Pharmacologia; or, the History of Medicinal Substances, with a view to establish the Art of Prescribing, and of composing extemporaneous Formulæ upon fixed and scientific Principles. By John Ayrton Paris, M.D. F.L.S. Fourth Edition. 8vo.

[MESSRS. HIGHLEY AND SON.]

